# Carrying a Pregnancy to Term With an Intrauterine Device in Place: A Case of an Intraabdominal Intrauterine Device Migration

**DOI:** 10.7759/cureus.63097

**Published:** 2024-06-25

**Authors:** Joshua J Oliver, Ethan P Kelly, Forest C Aller, Rachel E Bridwell, Justine K Stremick

**Affiliations:** 1 Department of Emergency Medicine, Madigan Army Medical Center, Tacoma, USA

**Keywords:** imaging modalities, unintended pregnancy, missed diagnosis, abdominal foreign body, migrated intrauterine device

## Abstract

Long-acting intrauterine contraceptives such as intrauterine devices (IUD) are popular due to their high rates of long-term efficacy, ease of use, and reversibility. Though rare, these devices can incur complications such as uterine perforation. Signs and symptoms are often vague abdominal and pelvic pain, and patients rarely present with a surgical emergency. This uterine perforation can happen immediately upon IUD placement or in a delayed manner. This case details an example of an IUD uterine perforation with abdominal migration two years after placement. The patient's history is complicated by the unique fact that she became pregnant and carried her pregnancy to a term vaginal delivery after the IUD had been placed. Her pregnancy led healthcare providers from previous encounters to believe that the IUD had been spontaneously expelled. The IUD was identified in the patient's left lower abdominal cavity via computed tomography (CT) and was surgically removed uneventfully.

## Introduction

Roughly 10 percent of females in the United States aged 15-49 use contraception in the form of long-acting intrauterine devices (IUDs) because they are highly effective, long-acting, and reversible [1, 2]. The mechanism of IUDs' contraceptive effect is to create an environment that is inhospitable to sperm within the cervix and uterus. However, for the device to be most effective, it has to be correctly seated within the fundus of the uterus [3]. IUDs incur a variety of potential complications. These range from mild and expected, such as abdominal cramping and abnormal uterine bleeding, to severe such as uterine perforation and pelvic inflammatory disease [1, 2]. While rare, uterine perforation is a recognized complication of IUD placement with an incidence of 1 per 1000 insertions [4]. Perforation typically occurs on initial insertion, though delayed perforation has been reported up to 42 years after insertion and is believed to be secondary to partial uterine wall perforation upon insertion, followed by erosion through the uterine wall over time [4]. While there is no data on the incidence of intra-abdominal IUD migration, numerous case reports describe IUD migration to the colon, bladder, and other intraperitoneal locations [5]. Complications of IUD migration depend on the exact location of the device but include abdominal pain, pelvic pain, bladder perforation, adhesions, bowel perforation, and bowel obstruction [4, 5]. Here, we present a unique case of delayed IUD migration.

## Case presentation

A 36-year-old female presented to the emergency department complaining of one week of undulating lower abdominal pain for one week, occasionally radiating to her back. The patient could not identify any inciting event associated with the onset of symptoms, specifically denying any preceding trauma. She did endorse mild dysuria and urgency but denied any fevers, chills, or abnormal vaginal bleeding or discharge. She is married and monogamous with her spouse. She presented with normal vital signs consisting of an oral temperature of 37.0 degrees Celsius, a heart rate of 89 beats per minute, a respiratory rate of 16 breaths per minute, a blood pressure of 118/64 millimeters of mercury, and pulse oximetry of 97 percent on room air. Physical exam showed mild suprapubic and uterine tenderness as well as mild cervical motion tenderness (CMT) with a normal appearing cervix and no abnormal vaginal discharge. No IUD strings were found on the exam. Testing in the Emergency Department revealed an unremarkable complete blood count with a normal white blood count, a normal complete metabolic panel, a normal urinalysis, a negative urine pregnancy test, and a negative vaginal smear. She was treated empirically for pelvic inflammatory disease with 500 milligrams (mg) of ceftriaxone once via intramuscular injection and 100 mg of doxycycline by mouth twice daily for 14 days. Gonorrhea and chlamydia testing resulted in negative several days following discharge.

**Figure 1:**
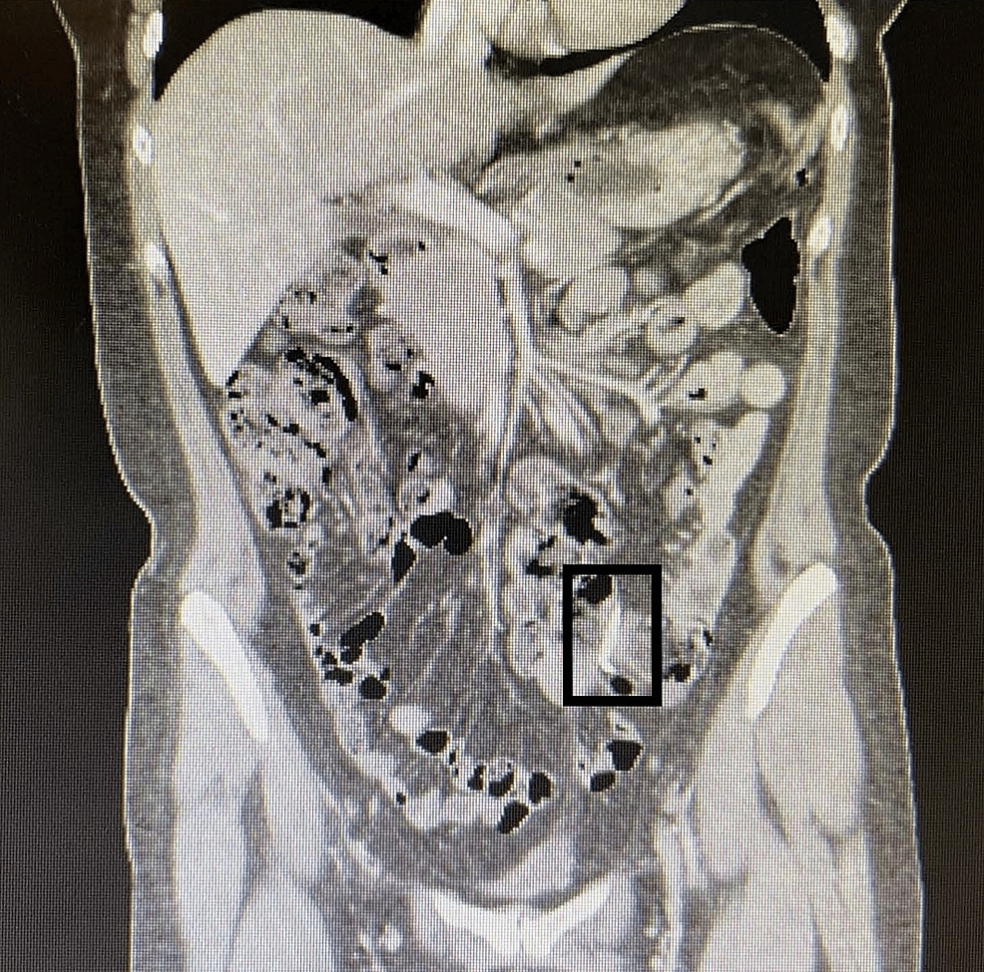
Computed tomography scan of the abdomen and pelvis without intravenous contrast demonstrating an extrauterine intrauterine device in the patient's left lower quadrant identified by a black rectangle

However, as the patient was being prepared for discharge, she asked if her pain could be related to her IUD. She went on to explain she had an IUD placed containing levonorgestrel two years previously, had subsequently become pregnant, and carried a healthy child to term via vaginal delivery. She also noted that she had inquired about the possibility of a retained IUD during previous healthcare encounters and been told that it was unlikely she could have become pregnant with an IUD in place, suggesting that it had spontaneously dislodged. As ultrasound (US) was not available overnight in the treating facility, a computed tomography (CT) scan of the abdomen and pelvis without contrast (Figure 1) demonstrated an intraabdominal IUD on the anterior abdominal wall in the left lower quadrant without associated abscess or fluid collection. Gynecologic surgery consultation was pursued, and the on-call physician advised outpatient evaluation, requesting placement of a referral as the patient was stable. Ultimately, the patient consented to operative laparoscopy and IUD removal. The intraoperative report described a retained IUD wrapped in the peritoneum without evidence of inflammation or damage to surrounding structures. The IUD was removed without incident, and the patient had a new levonorgestrel IUD placed. The patient made a full post-operative recovery without complications.

## Discussion

While IUDs can be spontaneously expelled, the rate of spontaneous expulsion has not been well studied; one retrospective study identified a rate of 11 percent [6]. Further, IUDs are a very effective form of birth control, with failure rates of 0.08 percent and 0.02 percent for copper-containing and levonorgestrel-containing IUDs, respectively [7]. Given these statistics, patients who present with concerns for potentially displaced IUDs with concern for uterine perforation, particularly those who are also pregnant, deserve further investigation as this is a strong indication that the IUD is not in the uterine fundus. In addition to the complications described above, these patients are also at an increased risk of ectopic pregnancy, which, in addition to uterine perforation, can significantly impact future fertility [4]. To prevent missing similar instances of retained perforated IUDs in the future, we recommend the following; assess the patient's vital signs and perform an abdominal examination to determine if the patient has signs and symptoms of sepsis or a peritoneal abdomen requiring antibiotics or urgent surgical consultation. 

IUD perforation is very rarely associated with sepsis, and this was much more common with older-generation IUDs that were more likely to cause bowel or bladder perforation [8]. In this instance, the patient required urgent surgical intervention; however, the majority of patients can be managed with minimally invasive surgery [9]. Further evaluation should be guided by the patient's presenting symptoms which are most commonly non-specific abdominal pain, vaginal bleeding, and an inability to find IUD strings on self-examination. Although our patient also had CMT. This makes abdominal and genitourinary examination a necessity to attempt to localize pain [9]. It should be noted if IUD strings are present in the cervical os; however, a case has been reported of uterine perforation with IUD strings present, meaning the IUD remained at least partially seated within the uterine fundus [10]. Even if IUD strings are identified, evaluation of uterine perforation and IUD migration should be pursued if clinical concern persists [10]. Transvaginal ultrasound (US) is the ideal first imaging modality to best visualize the IUD location if still intrauterine. If the US fails to identify an IUD, an abdominal X-ray is an option, but a CT of the abdomen and pelvis with intravenous contrast is the definitive imaging to locate the IUD and visualize potential complications, including abscess with loculations [4, 10]. IV contrast was not used in this case as infection was not suspected. If partial or complete uterine perforation is suspected or identified, a gynecology consultation is key. Historically, the expert opinion dictated surgical removal of any device that perforated the uterus [4]. However, with modern IUDs used in the United States, removal is not always necessary, as adhesions caused by IUD retrieval may cause more problems than the IUD itself, depending on the IUD's anatomical location, duration of perforation, and the patient's preferences. If the IUD is found not to be in the uterine fundus, alternative birth control should be offered to maintain effective birth control in the interim [3].

Disclaimer: the views expressed in this article are those of the authors and do not reflect the official policy of the Department of the Army, the Department of Defense, or the U.S. Government. The investigators have adhered to the policies for the protection of human subjects as prescribed in 45 CFR 46.

## Conclusions

While popular due to their efficacy and ease, IUDs rarely do cause issues ranging from abnormal uterine bleeding to uterine perforation with extra-uterine migration. This case highlights that if a patient raises a concern for a retained IUD, a history of pregnancy after IUD placement should increase clinical concern for uterine perforation.
